# A diagnostic pitfall in cold agglutinin disease: KMT2D-mutated CAD-associated lymphoproliferative disorder with a CLL-like immunophenotype

**DOI:** 10.1093/omcr/omag061

**Published:** 2026-05-10

**Authors:** Yusuke Okamoto, Hiroshi Arima, Kenichi Ishiyama, Kazuhisa Chonabayashi, Junya Kanda, Kouhei Yamashita, Masakazu Fujimoto, Hironori Haga, Yasuhito Nannya, Seishi Ogawa, Akifumi Takaori-Kondo

**Affiliations:** Department of Hematology, Graduate School of Medicine, Kyoto University, Yoshidakonoe-cho, Sakyo-ku, Kyoto 606-8303, Japan; Department of Hematology, Graduate School of Medicine, Kyoto University, Yoshidakonoe-cho, Sakyo-ku, Kyoto 606-8303, Japan; Department of Hematology, Graduate School of Medicine, Kyoto University, Yoshidakonoe-cho, Sakyo-ku, Kyoto 606-8303, Japan; Department of Hematology, Graduate School of Medicine, Kyoto University, Yoshidakonoe-cho, Sakyo-ku, Kyoto 606-8303, Japan; Department of Hematology, Graduate School of Medicine, Kyoto University, Yoshidakonoe-cho, Sakyo-ku, Kyoto 606-8303, Japan; Department of Hematology, Graduate School of Medicine, Kyoto University, Yoshidakonoe-cho, Sakyo-ku, Kyoto 606-8303, Japan; Department of Diagnostic Pathology, Kyoto University Hospital, 54 Shogoin-kawahara-cho, Sakyoku, Kyoto 606-8507, Japan; Department of Diagnostic Pathology, Kyoto University Hospital, 54 Shogoin-kawahara-cho, Sakyoku, Kyoto 606-8507, Japan; Department of Pathology and Tumor Biology, Graduate School of Medicine, Kyoto University, Yoshidakonoe-cho, Sakyo-ku, Kyoto 606-8303, Japan; Division of Hematopoietic Disease Control, Institute of Medical Science, The University of Tokyo, 4-6-1 Shirokanedai, Minato-ku, Tokyo 108-8639, Japan; Department of Pathology and Tumor Biology, Graduate School of Medicine, Kyoto University, Yoshidakonoe-cho, Sakyo-ku, Kyoto 606-8303, Japan; Institute for the Advanced Study of Human Biology (WPI-ASHBi), Kyoto University, Yoshidakonoe-cho, Sakyo-ku, Kyoto 606-8303, Japan; Department of Innovative Medicine, Faculty of Medicine, Kindai University, 377-2 Ono-Higashi, Osakasayama, Osaka 589-0014, Japan; Department of Hematology, Graduate School of Medicine, Kyoto University, Yoshidakonoe-cho, Sakyo-ku, Kyoto 606-8303, Japan

**Keywords:** cold agglutinin disease, CAD-associated lymphoproliferative disorder, KMT2D mutation

## Abstract

Cold agglutinin disease (CAD) is an uncommon hemolytic anemia by complement-fixing IgM autoantibodies and is frequently associated with a distinct clonal B-cell lymphoproliferative disorder (CAD-LPD). We report a woman in her 50’s with progressive fatigue who presented with hemolytic anemia and an IgM-κ paraprotein. Bone marrow examination revealed a small clonal B-cell population (5.8%) with a CLL-like immunophenotype. Despite the absence of peripheral lymphocytosis or established treatment indications for chronic lymphocytic leukemia (CLL), B-cell–directed therapy, including ibrutinib and venetoclax plus rituximab, was administered over the course of approximately one year based on the initial diagnostic impression, but failed to control hemolysis. Targeted next-generation sequencing identified a *KMT2D* frameshift mutation, supporting a diagnosis of CAD-LPD rather than monoclonal B-cell lymphocytosis. Subsequent treatment with the C1s inhibitor sutimlimab resulted in sustained resolution of hemolysis. This case highlights diagnostic pitfalls in CAD and underscores the value of molecular profiling in guiding appropriate therapy.

## Introduction

Cold agglutinin disease (CAD) is a rare form of autoimmune hemolytic anemia characterized by chronic hemolysis caused by cold-reactive, complement-fixing IgM autoantibodies that bind red blood cells at low temperatures and activate the classical complement pathway. In adults, CAD is now recognized as a clonal B-cell–driven disorder with IgM-κ paraprotein rather than a purely autoimmune condition. Most cases are associated with a distinct low-grade lymphoproliferative disorder, termed CAD-associated lymphoproliferative disorder (CAD-LPD), which is biologically and clinically separate from other mature B-cell neoplasms such as chronic lymphocytic leukemia (CLL) or lymphoplasmacytic lymphoma [1].

Recent molecular studies have refined this concept. Somatic mutations in lysine methyltransferase 2D (*KMT2D*) and caspase recruitment domain-containing protein 11 (*CARD11*) encoded proteins were identified in 11 of 16 (69%) and 5 of 16 (31%) patients with CAD, respectively [2]. Pathological examination of bone marrow from 54 CAD patients showed a small population (less than 10%) of aggregates of small lymphoid cells expressing the cell surface markers CD20^+^, IgMs^+^, CD27^+^, CD5^−/+^, and CD23^−^ in 40 patients [3].

Distinguishing CAD-LPD from other B-cell malignancies, such as CLL or monoclonal B-cell lymphocytosis (MBL) with a CLL phenotype can be challenging when marrow involvement is minimal and immunophenotypic overlap exists [4]. This distinction is nevertheless critical, as therapeutic strategies differ fundamentally. While B-cell–directed therapies are reserved for progressive or symptomatic CLL, complement inhibition has emerged as a highly effective and mechanism-based treatment for CAD. Sutimlimab, a humanized monoclonal antibody targeting complement C1s, has demonstrated rapid and sustained control of hemolysis and is now approved for CAD treatment in Japan [5].

We describe a patient initially managed as CLL based on immunophenotypic findings, in whom clinicopathological reassessment and molecular profiling led to reclassification as CAD-LPD and successful treatment with sutimlimab.

## Case report

A woman in her 50’s presented with progressive fatigue and jaundice occurring during colder seasons for approximately three years prior to presentation. She had no significant past medical history and no history of cytotoxic chemotherapy, immunosuppressive therapy, or recent infection. Physical examination revealed scleral icterus and mild splenomegaly without palpable lymphadenopathy. Cold-induced Raynaud’s phenomenon was noted.

Laboratory evaluation demonstrated normocytic anemia with a hemoglobin level of 10.5 g/dl (reference range 11.6–14.8 g/dl) and a markedly elevated reticulocyte count of 58.9‰ (reference range 7.0–20.0‰). The white blood cell (WBC) count was 9.23 × 10^9^/L with a normal absolute lymphocyte count of 3.29 × 10^9^/L. Lactate dehydrogenase and indirect bilirubin were mildly elevated, while serum haptoglobin was undetectable, consistent with active hemolysis. Complement levels (C3 and C4) were also low, and antinuclear antibodies and rheumatoid factor were negative. The soluble interleukin-2 receptor was slightly raised at 696 U/mL (reference range:121–613 U/mL) (Table 1). The direct antiglobulin test was strongly positive for C3d and negative for IgG. Cold agglutinin titers exceeded 1:8192 at 4°C. Serum protein electrophoresis revealed a monoclonal IgM-κ paraprotein.

**Table 1:** Laboratory test results

CBC			normal range
WBC	9.23	×10^9^ /L	3.3-8.6
Neutrophil	55.5	%	46.0-62.0
Lymphocyte	35.6	%	30.0-40.0
Monocyte	6.4	%	4.0-7.0
Eosinophil	1.7	%	3.0-5.0
Basophil	0.8	%	0.0-1.0
RBC	3.23	×10^12^ /L	4.35-5.55
Hb	10.5	g/dl	13.7-16.8
Hct	30.8	%	40.7-50.1
PLT	343	×10^9^ /L	158-348
reticulocyte	58.9	‰	6.7-18.1
Biochemistry			
AST	18	U/L	13-30
ALT	13	U/L	10-42
LDH	231	U/L	124-222
ALP	104	U/L	38-113
TP	6.7	g/dL	6.6-8.1
ALB	4.4	g/dL	4.1-5.1
ChE	213	U/L	240-486
TB	7.6	mg/dL	0.4-1.5
DB	<0.1	mg/dL	<0.2
Cre	0.56	mg/dL	0.65-1.07
UA	4.7	mg/dL	3.7-7.0
BUN	16	mg/dL	8-20
Na	138	mmol/L	138-145
K	4.6	mmol/L	3.6-4.8
Cl	106	mmol/L	101-108
Ca	9.2	mg/dL	8.8-10.1
P	3.9	mg/dL	2.7-4.6
CRP	<0.10	mg/dL	<0.14
IgG	890	mg/dL	861-1747
IgA	74	mg/dL	93-393
IgM	527	mg/dL	33-183
haptoglobin	<1	mg/dL	19-170
Ferritin	156	ng/mL	50-200
sIL-2R	696	U/mL	121-613

CBC: Complete Blood Count, WBC: White Blood Cell count, RBC: Red Blood Cell count, Hb: Hemoglobin, Hct: Hematocrit, PLT: Platelet count, AST: Aspartate Aminotransferase, ALT: Alanine Aminotransferase, LDH: Lactate Dehydrogenase, ALP: Alkaline Phosphatase, TP: Total Protein, ALB: Albumin, ChE: Cholinesterase, TB: Total Bilirubin, DB: Direct Bilirubin, Cre: Creatinine, UA: Uric Acid, BUN: Blood Urea Nitrogen, Na: Sodium, K: Potassium, Cl: Chloride, Ca: Calcium, P: Phosphate (Inorganic Phosphorus), CRP: C-Reactive Protein, IgG: Immunoglobulin G, IgA: Immunoglobulin A, IgM: Immunoglobulin M, sIL-2R: Soluble Interleukin-2 Receptor

**Figure 1:**
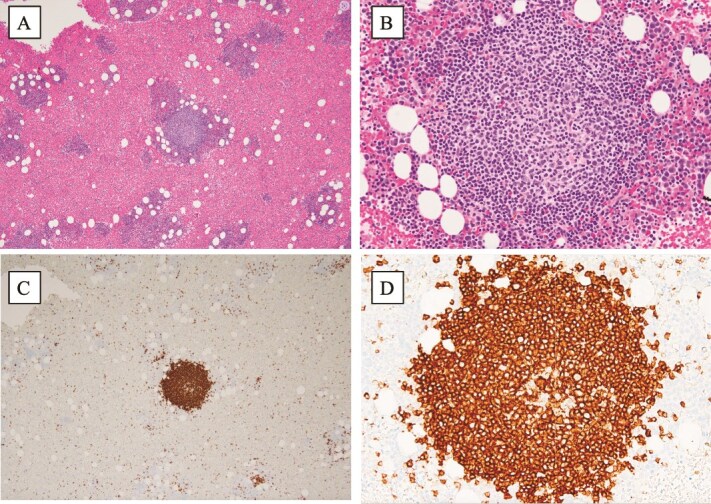
Histopathology in bone marrow biopsy specimen. A bone marrow biopsy demonstrates 80% cellularity with intraparenchymatous nodular B lymphoid lesions (A, ×40, and B, ×200, haematoxylin-eosin stain). These cells are positive for CD20 (C, ×40, and D, ×200).

Bone marrow biopsy showed hypercellularity with a small clonal B-cell population accounting for 5.8% of nucleated cells. These cells expressed CD19, CD20, CD5, partial CD23, surface IgM-κ, and HLA-DR. Histologically, intraparenchymal nodular lymphoid aggregates composed of small lymphocytes were observed with no morphological or immunophenotypic features suggestive of other low-grade B-cell lymphomas, including lymphoplasmacytic lymphoma (Fig. 1). Conventional cytogenetic analysis was normal.

Given the CLL-like immunophenotype, the initial diagnostic impression favored CLL, despite the absence of sustained peripheral lymphocytosis, cytopenias attributable to marrow infiltration, lymphadenopathy, or other standard indications for CLL therapy. Due to clinical urgency driven by severe, progressive hemolysis requiring frequent red blood cell transfusions, oral ibrutinib (420 mg/day) was initiated based on this assumption; however, hemolysis persisted despite two months of oral therapy. Subsequent treatment with venetoclax (100 mg/day) combined with rituximab (375 mg/m^2^ monthly) also failed to improve hemolytic parameters.

Targeted next-generation sequencing of bone marrow cells identified a heterozygous *KMT2D* frameshift mutation (c.10150_10160del, p.Thr3384fs) with a variant allele frequency of 5.3%; no pathogenic variants were detected in *MYD88* or *CARD11*. No features suggestive of infection, autoimmune systemic disease, or myelodysplastic syndrome were identified. Taken together—IgM paraproteinemia, complement-mediated hemolysis, minimal marrow infiltration with characteristic histology, presence of a *KMT2D* mutation, and refractoriness to B-cell–directed therapy—the diagnosis was revised to CAD-LPD.

Treatment was switched to complement inhibition by sutimlimab (6.5 g intravenously on days 0 and 7, followed by dosing every 14 days) with no adverse event. Hemoglobin levels gradually increased, markers of hemolysis normalized, and neutrophil and platelet counts recovered. At 12 months of follow-up, the patient remains clinically stable on maintenance sutimlimab without infectious complications.

## Discussion

This case illustrates a common diagnostic pitfall in CAD: minimal bone marrow involvement by a clonal B-cell population with a CLL-like immunophenotype can lead to misclassification as CLL or MBL with CLL phenotype. According to current diagnostic criteria, CLL requires sustained peripheral blood lymphocytosis ≥5 × 10^9^/L or cytopenias attributable to marrow infiltration, and MBL is defined as the presence of a clonal B-cell population below 5 × 10^9^/L in the peripheral blood, in the absence of lymphadenopathy [6]. These criteria were not met in our patient, making a diagnosis of overt CLL inappropriate.

Moreover, the presence of clinically significant complement-mediated hemolytic anemia is not adequately explained by MBL alone, supporting the interpretation that the marrow clone represented CAD-LPD rather than CLL or MBL with CLL phenotype. Taken together, CAD-LPD should be conceptualized as a bone marrow–based lymphoproliferative disorder with prominent systemic manifestations driven by complement activation, rather than a leukemic process defined by peripheral blood involvement.

Molecular findings were critical in resolving this ambiguity. *KMT2D* mutations are rare in CLL, reported in approximately 1% of cases in Western cohorts [7, 8], whereas they are detected in 30%–70% of CAD-LPD cases [2, 9, 10]. Although recent Asian studies suggest a somewhat higher prevalence of *KMT2D* mutations in CLL [11, 12], the marked enrichment of this alteration in CAD-LPD strongly supported reclassification in the present case. Notably, a previously reported Japanese case of cold agglutinin syndrome associated with *KMT2D*- and *CARD11*-mutated ‘CLL’ would also meet current criteria for CAD-LPD under the 5th edition of the WHO classification [13, 14].

Therapeutically, this distinction has major implications. Although B-cell–directed therapies, including BTK inhibitors, have been reported to be effective in a subset of patients with CAD [15, 16], their efficacy may depend on accurate characterization of the underlying clonal disorder. In the present case, BTK inhibition failed to control hemolysis, whereas targeted complement inhibition directly addressing the central pathogenic mechanism resulted in clinical benefit. Treatment with sutimlimab produced sustained hematologic improvement after failure of multiple prior B-cell–directed therapies, underscoring its role as a disease-modifying treatment for CAD.

Early recognition of CAD-LPD enables prompt initiation of complement blockade, potentially avoiding unnecessary exposure to cytotoxic or targeted B-cell therapies and reducing transfusion requirements [17, 18].
